# Seroprevalence of rickettsial infections and Q fever in Bhutan

**DOI:** 10.1371/journal.pntd.0006107

**Published:** 2017-11-27

**Authors:** Tshokey Tshokey, John Stenos, David N. Durrheim, Keith Eastwood, Chelsea Nguyen, Stephen R. Graves

**Affiliations:** 1 Faculty of Health and Medicine, University of Newcastle, Newcastle, Australia; 2 Australian Rickettsial Reference Laboratory, University Hospital Geelong, Geelong, Australia; 3 Department of Laboratory Medicine, Jigme Dorji Wangchuck National Referral Hospital, Thimphu, Bhutan; 4 Population Health, Hunter New England Local Health District, NSW Health, Newcastle, Australia; Mahidol Univ, Fac Trop Med, UNITED STATES

## Abstract

**Background:**

With few studies conducted to date, very little is known about the epidemiology of rickettsioses in Bhutan. Due to two previous outbreaks and increasing clinical cases, scrub typhus is better recognized than other rickettsial infections and Q fever.

**Methodology:**

A descriptive cross-sectional serosurvey was conducted from January to March 2015 in eight districts of Bhutan. Participants were 864 healthy individuals from an urban (30%) and a rural (70%) sampling unit in each of the eight districts. Serum samples were tested by microimmunofluorescence assay for rickettsial antibodies at the Australian Rickettsial Reference Laboratory.

**Results:**

Of the 864 participants, 345 (39.9%) were males and the mean age of participants was 41.1 (range 13–98) years. An overall seroprevalence of 49% against rickettsioses was detected. Seroprevalence was highest against scrub typhus group (STG) (22.6%) followed by spotted fever group (SFG) rickettsia (15.7%), Q fever (QF) (6.9%) and typhus group (TG) rickettsia (3.5%). Evidence of exposure to multiple agents was also noted; the commonest being dual exposure to STG and SFG at 5%. A person’s likelihood of exposure to STG and SFG rickettsia significantly increased with age and farmers were twice as likely to have evidence of STG exposure as other occupations. Trongsa district appeared to be a hotspot for STG exposure while Punakha district had the lowest STG exposure risk. Zhemgang had the lowest exposure risk to SFG rickettsia compared to other districts. People living at altitudes above 2000 meters were relatively protected from STG infections but this was not observed for SFG, TG or QF exposure.

**Conclusion:**

This seroprevalence study highlights the endemicity of STG and SFG rickettsia in Bhutan. The high seroprevalence warrants appropriate public health interventions, such as diagnostic improvements and clinical treatment guidelines. Future studies should focus on vector profiles, geospatial, bio-social and environmental risk assessment and preventive and control strategies.

## Author summary

Rickettsial infections including scrub typhus and Q fever are not widely recognised in Bhutan although the country is situated in an endemic Asian region. With two recorded outbreaks, scrub typhus is known to occur but other rickettsial infections and Q fever have not been recorded. In this first seroprevalence study of rickettsial infections, an overall seroprevalence of 49% was detected against rickettsioses amongst 864 participants. Seroprevalence was highest against scrub typhus group (STG) (22.6%) followed by spotted fever group (SFG) rickettsia (15.7%), Q fever (QF) (6.9%) and typhus group (TG) rickettsia (3.5%). Evidence of exposure to multiple agents were also noted; the commonest being dual exposure to STG and SFG at 5%. A person’s likelihood of exposure to STG and SFG significantly increased with age and farmers were twice as likely to have evidence of STG exposure as other occupations. Trongsa district in central Bhutan appeared to be a hotspot for STG exposure. People living at altitudes above 2000 meters were relatively protected from STG infections but this was not observed for SFG, TG and QF exposure. The findings from this study may direct future research on rickettsioses in Bhutan. These neglected tropical diseases warrant specific public health interventions in Bhutan.

## Introduction

Rickettsial infections including scrub typhus and Q fever are usually referred to as rickettsiosis [1]. Rickettsioses are zoonotic infections transmitted to humans through bites of infected ticks, fleas, lice and mites or through aerosols generated during exposure to infected placentas and birth fluids of mammals in the case of QF [2]. The family Rickettsiaceae includes two genera, *Rickettsia* and *Orientia*, which include many human pathogens some of which cause lethal infections with up to 30% mortality without treatment [3, 4]. The genus *Rickettsia* has more than 20 species making up several groups among which the spotted fever group (SFG) and typhus group (TG) are established human pathogens [4, 5]. The SFG rickettsia includes the etiologic agents of Rocky Mountain spotted fever (*R*. *rickettsii*) and Mediterranean spotted fever (*R*. *conorii*) and many others. The TG rickettsia include agents of epidemic (*R*. *prowazekii*) and endemic (*R*. *typhi*) typhus [4]. *Orientia* has two species; *O*. *tsutsugamushi* and *O*. *chuto* [6], together forming the scrub typhus group (STG). *Coxiella burnetii* is the causal agent of Q fever (QF). Of all the methods to detect rickettsial infections, antibody detection by serology is the most commonly used, microimmunofluorescence assay (IFA) being the currently accepted gold standard [7]. After an infection, IgM can be detectable for months and IgG for years [7, 8].

SFG and TG rickettsia occur worldwide and are a significant cause of morbidity in south-east Asia [9]. STG was originally thought to be confined to the Asia-Pacific region but now has been reported from the Middle East [6], Africa [10, 11] and South America [12]. Q fever has a worldwide distribution [13] except New Zealand [14] although fears of its introduction have been raised [15]. Rickettsioses are both emerging and re-emerging infections [16, 17]. Despite being endemic in Asia and causing significant burden to public health, true prevalence studies of these infections are limited. In India, rickettsial diseases including scrub typhus have been documented in several states from all parts of the country [1]. A seroepidemiology study in northeast India, in areas bordering Bhutan reported a sero-prevalence of 30.8%, 13.8% and 4.2% against STG, SFG and TG respectively [18]. In Darjeeling, another Indian district near Bhutan, a 2005 study reported an overall incidence of STG at 34 cases/100,000 population/pa, varying from 2 cases/100,000 population in July to 20/100,000 population in September and decreasing to zero in December [19]. Q fever has been under-reported from India and recent data are lacking [20]. A Chinse study reports an overall Q fever prevalence of 10% and highlights it as an under-reported and underdiagnosed illness [21].

Although situated in the endemic Asia Pacific region, Bhutan has reported scrub typhus cases only since 2009 [22] and SFG, TG and QF have not been reported to date. Rickettsial diseases (excluding Q fever) have been included in the national notifiable diseases since 2010 with increasing reports, mostly scrub typhus, from 118 cases in 2011 to 605 cases in 2015. Despite the increasing notifications and improving awareness, there are currently no clinical guidelines on management of rickettsial infections in Bhutan, and awareness needs improving. There are no reports of Q fever in Bhutan owing to the lack of diagnostic facility both in the human and animal sector at present. Therefore, a serological investigation was undertaken to determine the seroprevalence of rickettsial infections including QF in Bhutan.

## Methods

### Setting

**Fig 1:**
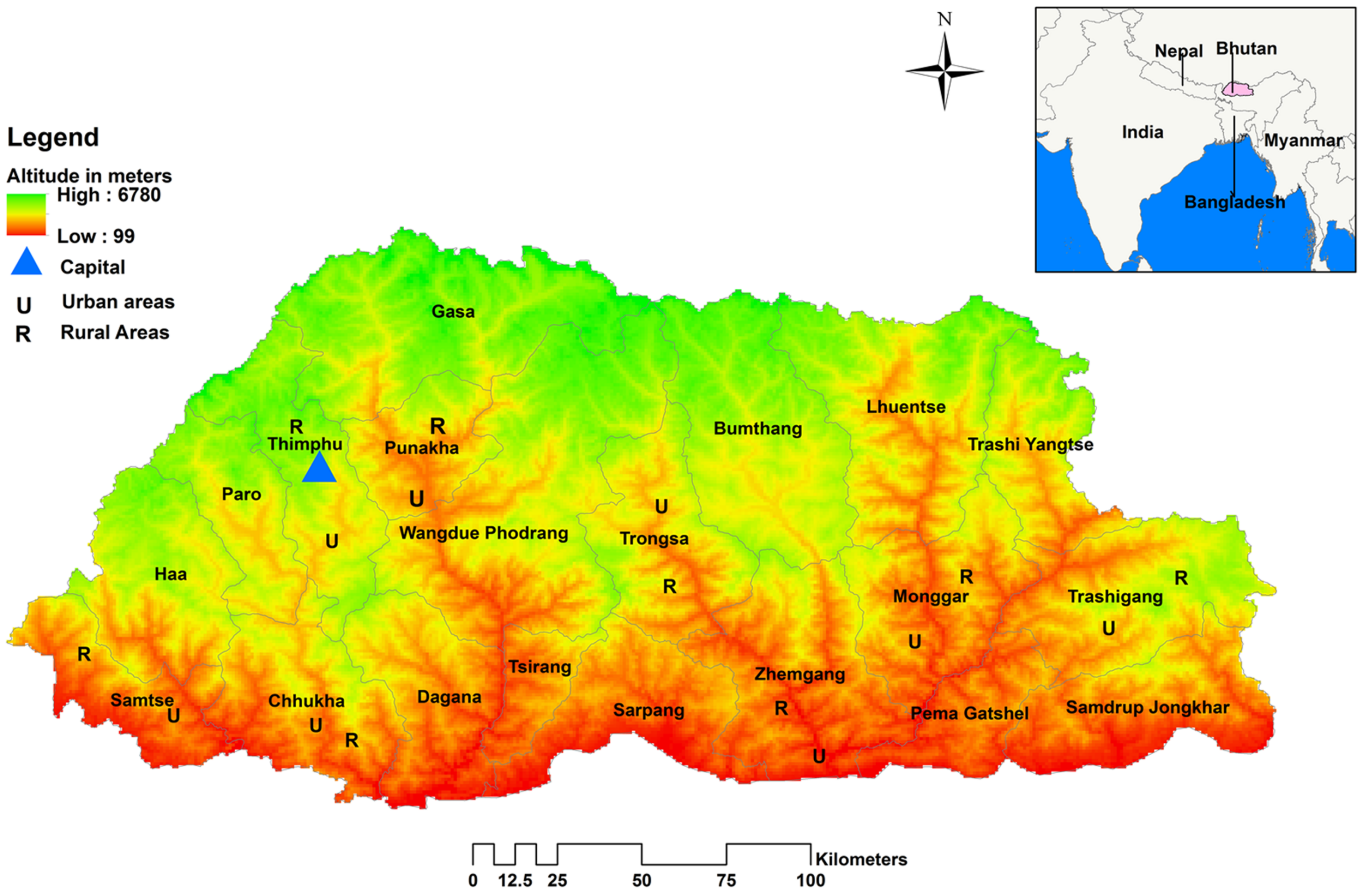
Map of Bhutan showing the eight selected districts with the location of urban (U) and rural (R) primary sampling units. (Map generated using the ESRI’s ArcMap 10.3.1 for desktop software. An electronic map of district boundaries of Bhutan in shapefile format was obtained from Global Administrative Areas database (http://www.gadm.org/country). Figure created solely for this manuscript and has not been used for any other publications or documents).

Bhutan is composed of 20 districts and 205 subdistricts with an estimated population of 770,000 in 2016 [23]. The Bhutan national census in 2005 reported on 1044 rural villages/chiwogs and 311 urban towns as primary sampling units (PSUs). Population density in different districts vary between 9–64 people/km^2^ [23]. For this study, the 20 districts were stratified into four regions; eastern (5 districts), central (4 districts), western (5 districts) and southern (6 districts) as defined by the Bhutan National Statistical Bureau (NSB) [23] for their national surveys. From each region, two districts were selected with a probability proportionate to size (PPS) method, selecting eight of twenty districts for the study (Fig 1). A rural and an urban PSU were selected from each district by the same PPS method. To assess the influence of altitude on exposure, altitude of places were arbitrarily grouped into low (<1000 meters), medium (1000–2000 meters) and high altitude (>2000 meters).

### Study design, participants and sample size

This descriptive cross-sectional sero-survey was carried out from January to March 2015, during the dry winter and early spring season. The sample size was calculated using a multi-stage cluster sampling method. Persons <13 years were excluded due to the possible risk of complications during blood sampling in remote areas. The sample size needed to estimate the number of households to be surveyed with a 95% confidence interval and other assumptions (50% prevalence rate, 0.05 margin of error, a design effect of 2 and an expected rate of participation of 90%) was calculated to be 864. Based on Bhutan’s urban-rural population proportion of 30:70 [23], 30% of the participants were taken from urban and the remaining from rural settings; therefore, of the 864 households selected, 256 were from urban and 608 from rural settings. Each of the eight selected districts contributed 108 households (76 rural and 32 urban households). The households were taken from the household list with unique identification numbers developed during the previous national surveys (National Health Survey 2012 and NCD STEPS Survey 2014). When a selected PSU had a lesser number of households than required, a nearby PSU was added. After selection of the household, all eligible members (≥13 years) present in the house were listed and one member was selected for the study through a lottery system. After selection, written consent was obtained; demographic details and environmental exposure history were taken by trained laboratory personnel through a face-to-face interview and blood samples were collected. Serum was extracted and stored at -70°C until shipment to Australia.

### Serological testing

Serum samples were shipped at room temperature to the Australian Rickettsial Reference Laboratory (ARRL) [24], a nationally accredited laboratory for rickettsial testing, where serological testing was carried out by indirect microimmunofluorescence assay (IFA) [25]. Antibodies against SFG rickettsia were individually tested using *R*. *australis*, *R*. *honei*, *R*. *conorii*, *R*. *africae*, *R*. *rickettsii* and *R*. *felis* antigens; TG rickettsia using *R*. *prowazekii* and *R*. *typhi* antigens; STG using *O*. *tsutsugamushi* (Gilliam, Karp and Kato strains) and *O*. *chuto* antigens, and QF using *C*. *burnetii* phase I and phase II antigens. Samples were initially screened at low dilutions and titrated to end-point (titre) when positive. With slight modification from the usual ARRL interpretation criteria [24, 25], antibody titres of ≥1:256 for IgG and/or ≥1:1024 for IgM against any of the SFG, TG and STG antigens were considered positive for the rickettsial group agents. Similarly an antibody titre of ≥1:50 for IgG or IgA and ≥1:100 against IgM against *C*. *burnetii* phase I or II or both were considered positive for Q fever. Positive and negative control wells were included in each slide during testing.

### Statistical analysis

Data were entered into an Excel spreadsheet and analysed using STATA software version 14. Chi-squared or Fischer’s exact test was used to explore the association between seropositivity and study variables considering p values of ≤0.05 significant. Univariate logistic regression was used to determine crude odds ratio (COR) and p values. All variables with p values 0.2 or less in the univariate analysis were taken for multivariate logistic regression to determine adjusted OR (AOR) and corresponding p values of <0.05 considered significant.

### Ethics and consent

The study was approved by the Bhutan Research Ethics Board of Health (REBH) (Ref: REBH/Approval/2014/019) and the Human Research Ethics Committee (HREC), University of Newcastle, Australia (Ref: H-2016-0085). All individuals or parent/guardian provided written consent before participation.

## Results

### Demography

A total of 864 participants were enrolled from the eight districts and all selected candidates consented to the study. There were 345 (39.9%) males and the mean age of participants was 41.1 (range 13–98) years. Most participants belonged to the age group of 26–40 years. Farmers 414 (47.9%) were the highest group by occupation (Table 1).

**Table 1:** Participants distribution by gender, occupation and location in different age groups (N = 864).

Variable	Age group (years)				
	13–25	26–40	41–55	>55	Overall (%)
**Total (%)**	163 (19)	311 (36)	210 (24)	180 (21)	864 (100)
**Gender**					
Male	59	112	97	77	345 (40)
Female	104	199	113	103	519 (60)
**Occupation**					
Farmers	63	113	114	124	414 (48)
Herders	1	13	16	11	41 (5)
Employees	17	87	32	8	144 (17)
Students	45	0	0	0	45 (5)
Housewives	21	90	37	34	182 (21)
Unemployed	16	8	11	3	38 (4)
**Sampling unit**					
Urban	39	121	52	44	256 (30)
Rural	124	190	158	136	608 (70)
**Altitude**					
Low	28	54	36	22	140 (16)
Medium	106	213	139	126	584 (68)
High	29	44	35	32	140 (16)

### Serology

**Fig 2:**
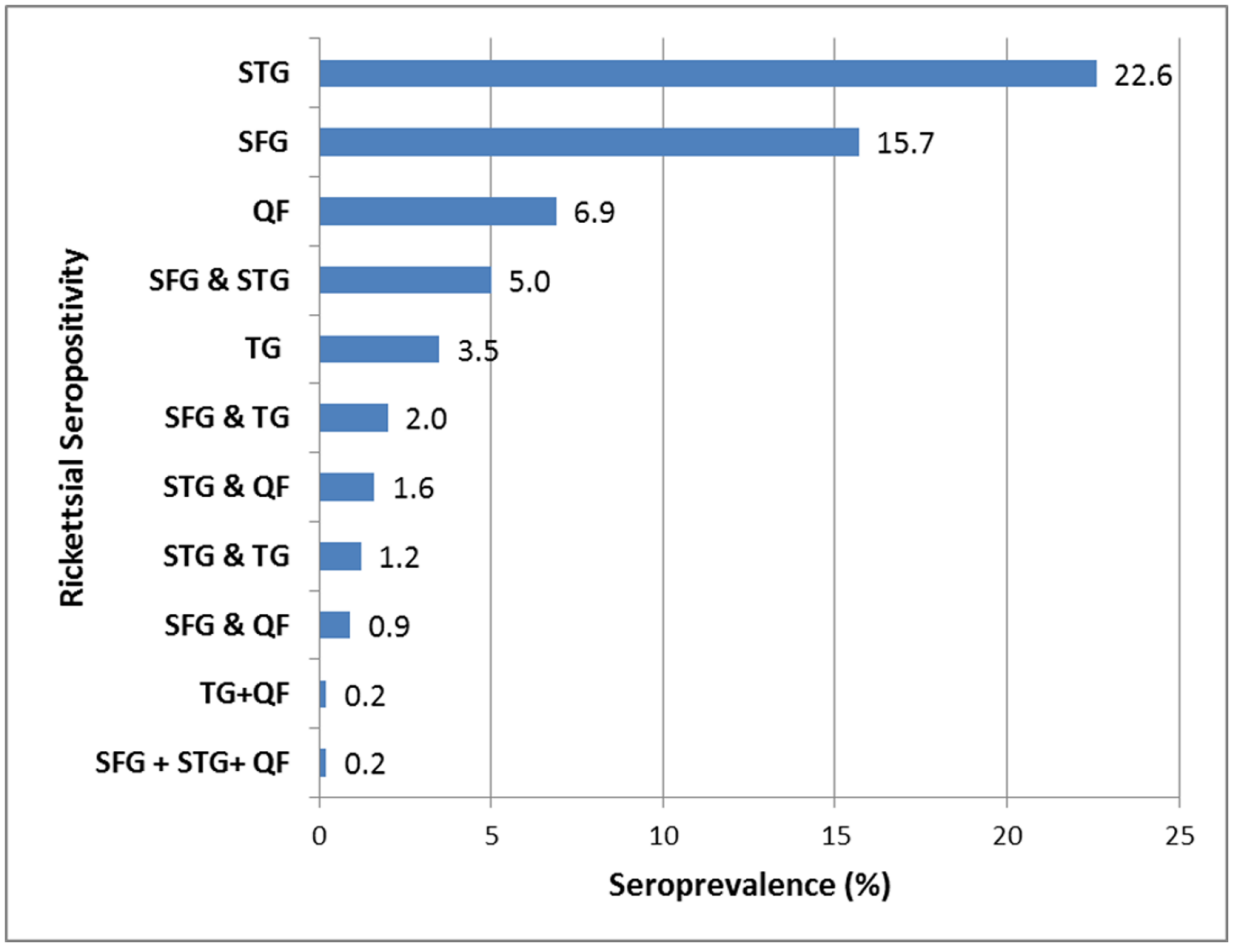
Overall seroprevalence of rickettsial infections in Bhutan. (STG, Scrub Typhus Group; SFG, Spotted Fever Group; TG, Typhus Group; QF, Q Fever).

In seropositive participants, most had IgG antibody titres of 1:256 or 1:512 against STG, SFG and TG rickettsia and titres of 1:100 or 1:200 against *Coxiella* phase II IgG, IgA or IgM. A very small number of high antibody titres of up to 1: 2048 in STG (≈3%) and SFG (≈ 0.1%) were seen in participants. Overall, the most prevalent rickettsial infection was STG (22.6%) and the least prevalent was TG rickettsia (3.5%). Evidence of past exposure to multiple agents was also seen; the commonest being dual exposure to SFG and STG (5%) (Fig 2).

Seropositivity rates were not significantly different between males and female for all four infectious agents. The prevalence of each infection, especially STG and SFG, appeared to increase with age and farmers exhibited the highest seropositivity rates.

### Sociodemographic and environmental determinants of exposure risk

Thirty percent (256) of the participants were from urban areas. Among all participants, 550 (63.7%) reported having animal contact and almost half (426) had pets at home. In addition, 620 (71.8%) reported contact with vegetation and forest during their daily activities, 205 (24.3) recollected suffering from febrile illness in the past, 337 (40.8%) had past tick bites, 153 (18.0%) had an eschar in the past and 202 (23.6%) had past flea bites.

Many of the demographic and environmental variables showed significant baseline correlation with seropositivity against each infection in Chi-squared or Fisher’s exact test (Table 2) and a few were statistically significant in the logistic regression analysis.

**Table 2:** Baseline correlation of seropositivity with different variables.

Variables	STG		SFG		TG		QF	
	Positive	*p* value	Positive	*p* value	Positive	*p* value	Positive	*p* value
**Gender**								
Male (n = 345)	76 (22.0)	0.757	54 (15.6)	0.954	9 (2.6)	0.258	25 (7.2)	0.776
Female (n = 519)	119 (22.9)		82 (15.8)		21 (4.0)		35 (6.7)	
**Age groups (yrs)**								
13–25 (n = 163)	23 (14.1)	0.001*	13 (8.0)	0.003*	5 (3.1)	0.107	12 (7.4)	0.619
26–40 (n = 311)	61 (19.6)		45 (14.5)		6 (1.9)		17 (5.5)	
41–55 (n = 210)	56 (26.7)		39 (18.6)		8 (3.8)		16 (7.6)	
> 55 (n = 180)	55 (30.6)		39 (21.7)		11 (6.1)		15 (8.3)	
**Occupation**								
Farmers (n = 414)	122 (29.5)	<0.001*	79 (19.1)	0.031*	16 (3.9)	0.14	29 (7.0)	0.103
Herders (n = 41)	8 (19.5)		5 (12.2)		3 (7.3)		6 (14.6)	
Office workers (n = 144)	14 (9.7)		15 (10.4)		2 (1.4)		5 (3.5)	
Students (n = 45)	3 (6.7)		2 (4.4)		0 (0.0)		3 (6.7)	
Housewives (n = 182)	39 (21.4)		31 (17.0)		6 (3.3)		12 (6.6)	
Unemployed (n = 38)	9 (23.7)		4 (10.5)		3 (7.9)		5 (13.2)	
**Districts**								
Chukha (n = 108)	32 (29.6)	<0.001*	29 (26.9)	0.006*	6 (5.6)	0.009*	9 (8.3)	0.034*
Mongar (n = 108)	13 (12.0)		13 (12.0)		2 (1.9)		13 (12.0)	
Punakha (n = 108)	10 (9.3)		18 (16.7)		10 (9.3)		3 (2.8)	
Samtse (n = 108)	25 (23.1)		22 (20.4)		2 (1.9)		2 (1.9)	
Thimphu (n = 108)	5 (4.6)		8 (7.4)		3 (2.8)		12 (11.1)	
Trashigang (n = 108)	19 (17.6)		18 (16.7)		5 (4.6)		6 (5.6)	
Trongsa (n = 108)	46 (42.6)		14 (13.0)		2 (1.9)		8 (7.4)	
Zhemgang (n = 108)	45 (41.7)		14 (13.0)		0 (0.00)		7 (6.5)	
**Sampling units**								
Rural (n = 608)	159 (26.2)	<0.001*	106 (17.4)	0.035*	26 (4.3)	0.065	41 (6.7)	0.72
Urban (n = 256)	36 (14.1)		30 (11.7)		4 (1.6)		19 (7.4)	

*p<0.05

**Fig 3:**
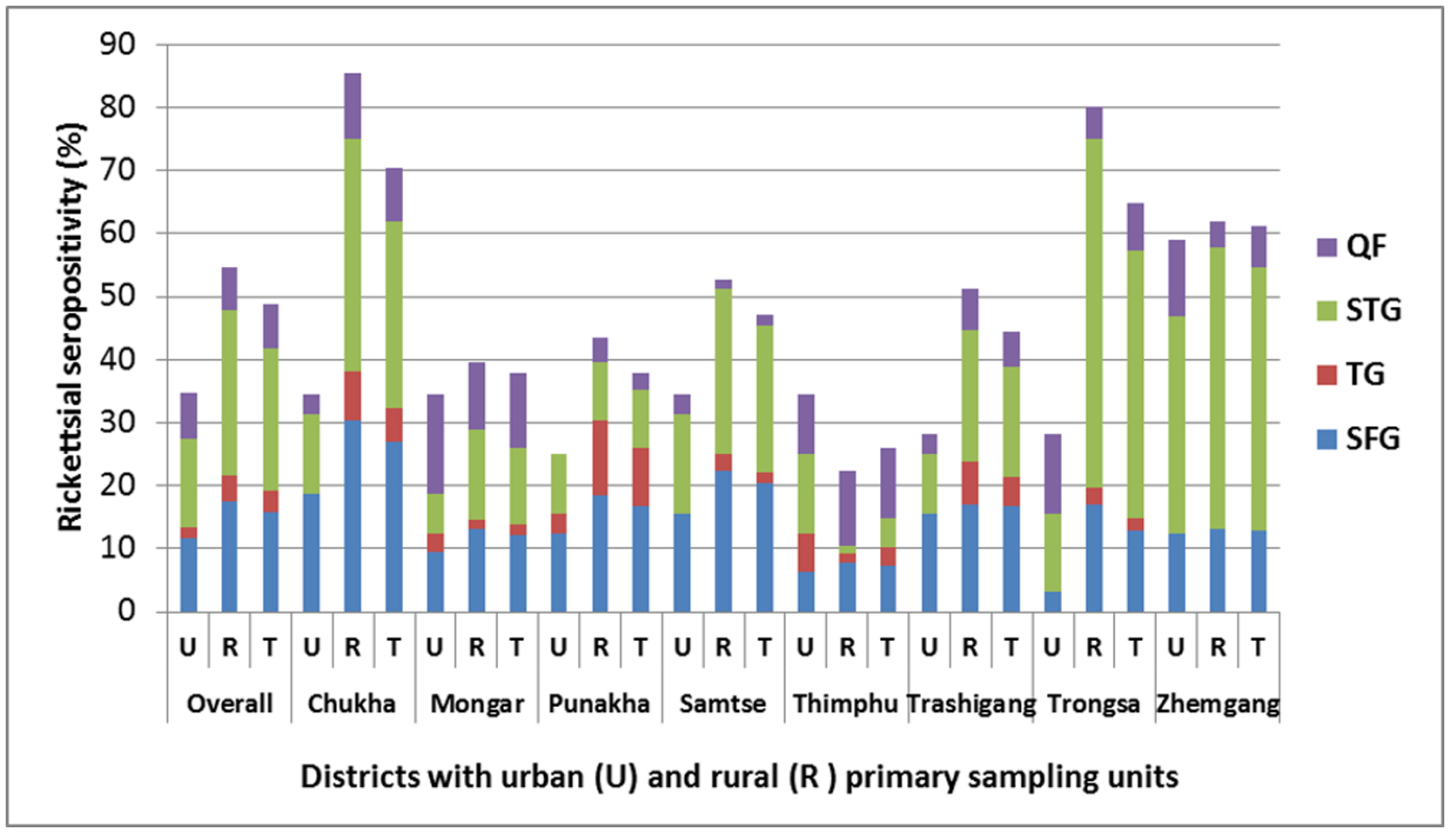
Prevalence of rickettsial seropositivity in different districts of Bhutan. (U, Urban; R, Rural; T, Total).

**Fig 4:**
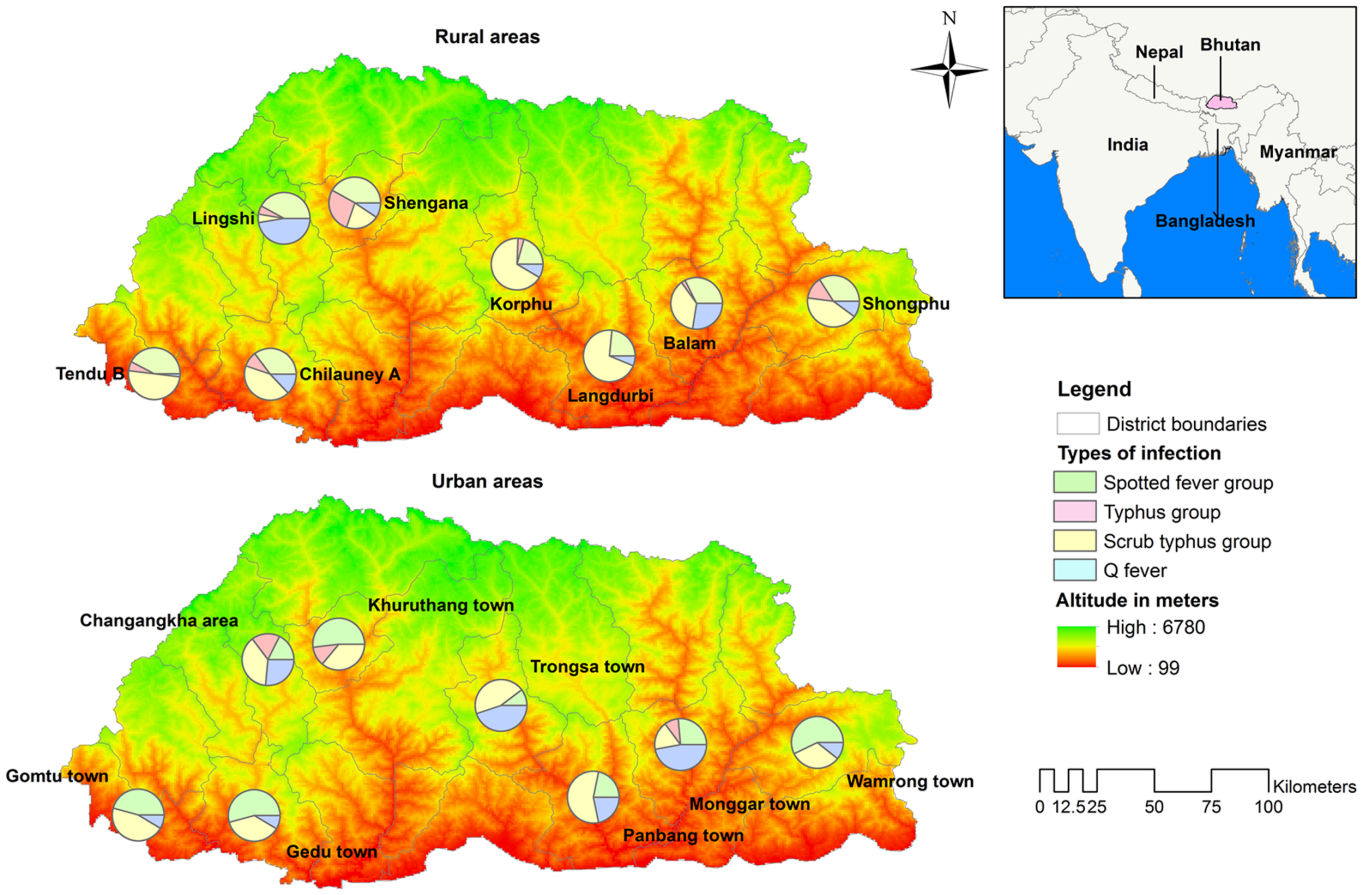
Prevalence estimates of the four infections in the urban and rural sampling units of the eight districts in Bhutan. (Map generated using the ESRI’s ArcMap 10.3.1 for desktop software. An electronic map of district boundaries of Bhutan in shapefile format was obtained from Global Administrative Areas database (http://www.gadm.org/country). Figure created solely for this manuscript and has not been used for any other publications or documents).

The comparative seropositivity in the urban and rural sampling units of the eight districts and the overall national prevalence of all four infections are shown in Fig 3 and the estimated proportion of each infection at different sampling units (urban and rural) of the eight districts presented in Fig 4. Significant epidemiological factors and seropositivity are shown for STG (Table 3) and SFG (Table 4). No factors showed association with QF or TG rickettsia seropositivity, likely due to the small number of seropositives.

**Table 3:** Epidemiological factors associated with seropositivity to Scrub typhus group (STG) rickettsioses in Bhutan.

Variables	COR	95% CI		*p* value	AOR	95% CI		*p* value
**Age group (years)**								
13–25	Ref.							
26–40	1.49	0.88	2.50	0.138	1.91	1.01	3.63	0.048*
41–55	2.21	1.29	3.79	0.004*	2.37	1.24	4.54	0.009*
> 55	2.68	1.56	4.61	<0.001*	3.14	1.59	6.20	0.001*
**Occupation**								
Office workers	Ref.							
Herders	0.58	0.26	1.29	0.183	1.94	0.62	6.09	0.254
Farmers	0.26	0.14	0.47	<0.001*	2.43	1.13	5.20	0.023*
Students	0.17	0.05	0.56	0.004*	1.26	0.29	5.47	0.755
Housewives	0.65	0.43	0.99	0.043	1.97	0.94	4.13	0.074
Unemployed	0.74	0.34	1.62	0.453	2.26	0.76	6.75	0.144
**Districts**								
Chukha	Ref.							
Mongar	0.33	0.16	0.66	0.002	0.42	0.17	1.02	0.056
Punakha	0.24	0.11	0.52	<0.001*	0.21	0.08	0.54	0.001*
Samtse	0.72	0.39	1.31	0.281	0.82	0.40	1.68	0.584
Thimphu	0.12	0.04	0.31	<0.001*	0.96	0.17	5.33	0.962
Trashigang	0.51	0.27	0.97	0.039	0.75	0.32	1.72	0.493
Trongsa	1.76	1.00	3.09	0.048*	3.44	1.53	7.74	0.003*
Zhemgang	1.70	0.97	2.98	0.066	1.63	0.81	3.28	0.174
**Altitude**								
Low	Ref.							
Medium	0.70	0.47	1.05	0.085	0.84	0.45	1.57	0.577
High	0.15	0.07	0.32	<0.001*	0.11	0.03	0.44	0.002*
**Animal contact**								
No	Ref.							
Yes	1.66	1.17	2.36	0.005	1.68	1.09	2.58	0.018

COR, crude odds ratio; AOR, adjusted odds ration;

*p<0.05

**Table 4:** Epidemiological factors associated with seropositivity to Spotted fever group (SFG) rickettsiosis in Bhutan.

Variables	COR	95% CI		*p* value	AOR	95% CI		*p* value
**Age group (years)**								
13–25	Ref.							
26–40	1.95	1.02	3.73	0.043*	1.68	0.81	3.48	0.159
41–55	2.63	1.35	5.12	0.004*	2.16	1.04	4.48	0.040*
> 55	3.19	1.64	6.23	0.001*	2.87	1.35	6.09	0.006*
**Districts**								
Chukha	Ref.							
Mongar	0.37	0.18	0.77	0.007*	0.46	0.19	1.14	0.094
Punakha	0.54	0.28	1.06	0.072	0.53	0.22	1.24	0.141
Samtse	0.70	0.37	1.31	0.263	0.7	0.34	1.47	0.348
Thimphu	0.22	0.09	0.50	<.001*	1.78	0.16	19.28	0.634
Trashigang	0.54	0.28	1.06	0.072	0.68	0.29	1.61	0.386
Trongsa	0.41	0.20	0.82	0.012*	0.62	0.25	1.54	0.301
Zhemgang	0.41	0.20	0.82	0.012*	0.34	0.15	0.77	0.010*
**Altitude**								
Low	Ref.							
Medium	0.66	0.42	1.03	0.067	0.82	0.43	1.59	0.564
High	0.23	0.11	0.51	<0.001*	0.11	0.01	1.07	0.058

COR, crude odds ratio; AOR, adjusted odds ration;

*p<0.05

The prevalence of STG seropositivity increased with age. The odds of exposure to STG infection was significantly higher in farmers compared to other occupations. Punakha district had the lowest risk of exposure to STG infections while people living in Trongsa district were three times more likely to be infected as those in other districts. Contact with domestic animals more than doubled the odds of exposure to STG. People residing at high altitude had 89% lower odds of being exposed to STG compared to those residing at lower altitude (AOR 0.11, p 0.002, 95% CI 0.03, 0.44), (Table 3).

SFG rickettsial seropositivity prevalence also increased with age. A person over 55 years of age was three times more likely to have been exposed to SFG than the younger age groups. Compared to other districts, Zhemgang district had a significantly lower odds of exposure to SFG rickettsia (AOR 0.34, p value = 0.010, 95% CI 0.15, 0.77). Altitude did not affect the prevalence of SFG (Table 4).

## Discussions

This study revealed an overall seroprevalence of 48.7% against rickettsioses in Bhutan with the highest prevalence to scrub typhus (22.6%) followed by SFG rickettsia (15.7%), Q fever (6.9%) and TG rickettsia (3.5%). Evidence of past exposure to two or more rickettsial agents was seen in 11.1% of the participants depicting probable dual or multiple infections in an endemic setting or possibly cross-reacting antibodies. This is the first seroprevalence study on rickettsioses in Bhutan and may be used as baseline data for subsequent studies in this country although it is recognised that prevalence estimates may vary when measured at different times of the year. The limitations of the findings from the exclusion of children (<13 years old) should be borne in mind. This was required to avoid risks of complications during blood sampling especially in remote areas where medical assistance is hard to obtain. Unintended but unavoidable exclusion of potential participants could have also occurred due to a member of the household being away from home during sampling. Information on past fevers, tick bites and eschar might have had drawbacks due to participants failing to comprehend technical terms. In addition, inter-district, urban versus rural as well as high versus low altitudes comparisons was not precise due to the highly variable landscape within and between districts. Unavailability of adequate local data on climatic conditions, environmental and geospatial information at primary sampling unit (urban and rural) level made it impossible to explain inter-district differences of exposure to the infections.

The findings in this study were similar to a seroprevalence study in north-east India, that reported the highest seroprevalence against STG (30.8%) followed by SFG (13.8%) and TG (4.2%) [18]. The similarity is noteworthy due to the proximity of these areas to Bhutan. Similar occurrences of these infections in neighbouring countries may benefit from coordinated cross-border prevention and control activities.

The odds of exposure sequentially increased with increasing age of participants in case of STG and SFG rickettsiae. This mirrors the situation in endemic areas where increasing number of people would be exposed to the infections as they advance through life leading to an accumulation of older seropositive people in the community. In south-east Asia, murine typhus was reported more in urban dwellers, while STG and SFG were more prevalent in rural dwellers [9]. However, in Bhutan, this study did not find any significant differences in any of the four infections between urban and rural residents probably reflecting similar environmental conditions between the two populations. This is supported by finding no significant differences between occupational groups for all infections, with the exception of STG where farmers had higher seropositivity rates compared to other occupations. There were differences between a few districts for STG and SFG infections, highlighting hot spots for these two infections. Trongsa district in central Bhutan appeared to be a hotspot for STG infection and Punakha district exhibited significantly low odds of exposure. STG exposures were significantly low amongst participants in high altitude areas. This may be explained by cold weather at high altitude areas not favouring mite survival. Of all the districts, Zhemgang in south-central Bhutan had the lowest odds of exposure to SFG rickettsia. Unlike STG infections, altitude had no effect on SFG, TG and QF exposure. This could be due to different tick species at different altitudes transmitting different infections. Expanding primary and secondary clusters of STG infections were also reported in China [26]. Such clusters or hotspots would benefit from focused public health interventions especially where resources are limited as in the case of Bhutan. Targeting prevention and control activities in hotspot areas could be effective and cost saving.

Antibody titres of 1:256 or 1:512 were the commonest observed antibody levels amongst the participants. A small number of participants with higher antibody titres of 1:1024 or 1:2048 may have been due to recent infections (symptomatic or asymptomatic) or due to recurrent subacute exposures stimulating antibody production. Cross-reactions between antibodies within the rickettsial species, especially between SFG and TG rickettsia, are known to occur. Therefore, persons with mixed antibodies may not necessarily have had multiple infections but may be due to cross-reacting antibodies resulting from the one infection. This could also explain some of the observed multi-species exposures. Background antibody levels in endemic situations are known to interfere with serological diagnosis of acute infections especially with rapid point-of-care diagnostics [17]. This is worthy of note in the Bhutanese setting where only rapid point-of-care diagnostics are available currently. There is urgent need to improve diagnostic facilities in Bhutan to provide more specific assays such as the microimmunofluorescence assay and molecular diagnostics, especially in the main centres. Point-of-care diagnostics could still be useful in the smaller districts and remote health centres for ease of use.

Rickettsioses have been associated with poor maternal and neonatal outcomes including stillbirth and low birth weights [27, 28] in endemic situations. The role of rickettsioses in the high maternal and neonatal mortality and morbidities in Bhutan deserves to be studied. Scrub typhus has also been known to involve the central nervous system manifesting as meningoencephalitis [29–31] and STG has been recently reported as a significant cause of encephalitis in northeast India [32]. This is important in the Bhutanese context in light of establishing the causes of acute encephalitis syndromes including meningococcal infection, Japanese encephalitis and other viral meningitis syndromes which are poorly understood at present. Documented deaths in Bhutan due to proven scrub typhus had resulted from meningoencephalitis and gastrointestinal perforation [22], which are known to be severe complications of scrub typhus. Understanding these occurrences in endemic areas could be helpful in averting preventable deaths from such complications.

Rickettsioses are important causes of illnesses in international travellers [33]. In a study on the spectrum of illness amongst ill returned travellers from six GeoSentinel sites, rickettsial infections were significant causes with 17 and 32 patients/1000 cases returning from south central and south-east Asia respectively [34]. Bhutan is an emerging destination for international travellers involving in activities like camping, trekking, cultural and rural home-stays. The high prevalence of rickettsioses could potentially expose travellers to these infections. Therefore travellers should be aware of the risk and become educated on preventive measures. In addition, educating travellers would keep them vigilant for any febrile illnesses during travel or upon returning to their home countries, enabling them to provide a detailed travel history and for their treating doctor to include rickettsial infections in their differential diagnoses.

There are limited prevalence studies on rickettsioses in south-east Asia [9]. Studies are even scantier in south and central Asia including Bhutan where most published studies were focused on clinical cases and acute febrile patients. Therefore, a prevalence study of these neglected but re-emerging infections in these endemic areas should be carried out with active regional collaborations and participations. This first seroprevalence study in Bhutan highlighted the endemicity of rickettsioses especially STG and SFG rickettsia. Findings on TG rickettsia and Q fever should be interpreted with caution due to the detection of fewer positive cases. This high rickettsial seroprevalence needs attention from the Bhutan Ministry of Health such as appropriate public health interventions, diagnostic improvement and clear clinical treatment guidelines. Future studies should focus on vector profiles, geospatial, bio-social and environmental risk assessment and preventive and control strategies formulation.

## Supporting information

S1 Metadata (XLSX)

S1 STROBE Checklist (DOC)
