# Supplementary material for: Seroprevalence of rickettsial infections and Q fever in Bhutan
Source: PLoS Negl Trop Dis. 2017 Nov 27;11(11):e0006107. doi: 10.1371/journal.pntd.0006107 (PMC5720829; doi:10.1371/journal.pntd.0006107)
Supplement: S1 STROBE Checklist — (DOC) [file pntd.0006107.s002.doc]

STROBE Statement—Checklist of items that should be included in reports of ***cross-sectional studies***

|  | Item No | Recommendation |
| --- | --- | --- |
| **Title and abstract** | 1 | (*a*) Indicate the study’s design with a commonly used term in the title or the abstract  **[Indicated within the title in page 1 and method section of the abstract page 2]** |
|  |  | (*b*) Provide in the abstract an informative and balanced summary of what was done and what was found **[Provided in method and results section of abstract on page 2]** |
| Introduction | | |
| Background/rationale | 2 | Explain the scientific background and rationale for the investigation being reported  **[Explained in the introduction section of manuscript on page 4]** |
| Objectives | 3 | State specific objectives, including any prespecified hypotheses **[Stated at the end of the introduction part of manuscript on page 4]** |
| Methods | | |
| Study design | 4 | Present key elements of study design early in the paper **[Presented under methods section of the manuscript on page 5]** |
| Setting | 5 | Describe the setting, locations, and relevant dates, including periods of recruitment, exposure, follow-up, and data collection **[Described under methods section of the manuscript on page 5-6]** |
| Participants | 6 | (*a*) Give the eligibility criteria, and the sources and methods of selection of participants **[Given under methods section on page 5-6]** |
| Variables | 7 | Clearly define all outcomes, exposures, predictors, potential confounders, and effect modifiers. Give diagnostic criteria, if applicable **[Defined under methods section on pages 5-6, diagnostic details provided in serological testing under methods page 6]** |
| Data sources/ measurement | 8* | For each variable of interest, give sources of data and details of methods of assessment (measurement). Describe comparability of assessment methods if there is more than one group **[Described under participants and statistical analysis section of methods, pages 5-7]** |
| Bias | 9 | Describe any efforts to address potential sources of bias **[Described under study design, participants and sample size section of methods, page 5-6]** |
| Study size | 10 | Explain how the study size was arrived at **[Explained in sample size under methods, page 5-6]** |
| Quantitative variables | 11 | Explain how quantitative variables were handled in the analyses. If applicable, describe which groupings were chosen and why **[Explained in methods, statistics page 5-6]** |
| Statistical methods | 12 | (*a*) Describe all statistical methods, including those used to control for confounding **[Described in statistics page 6]** |
|  |  | (*b*) Describe any methods used to examine subgroups and interactions **[Described in statistics page 6-7]** |
|  |  | (*c*) Explain how missing data were addressed **[Described in statistics page 6-7]** |
|  |  | (*d*) If applicable, describe analytical methods taking account of sampling strategy **[NA]** |
|  |  | (*e*) Describe any sensitivity analyses **[N/A]** |
| Results | | |
| Participants | 13* | (a) Report numbers of individuals at each stage of study—eg numbers potentially eligible, examined for eligibility, confirmed eligible, included in the study, completing follow-up, and analysed **[Reported in results section page 7]** |
|  |  | (b) Give reasons for non-participation at each stage **[N/A]** |
|  |  | (c) Consider use of a flow diagram **[N/A]** |
| Descriptive data | 14* | (a) Give characteristics of study participants (eg demographic, clinical, social) and information on exposures and potential confounders **[Given in results section page 7-9]** |
|  |  | (b) Indicate number of participants with missing data for each variable of interest **[Indicated in Sociodemographic and environmental determinants of exposure risk under results, page 9]** |
| Outcome data | 15* | Report numbers of outcome events or summary measures **[Reported in results section pages 9-11]** |
| Main results | 16 | (*a*) Give unadjusted estimates and, if applicable, confounder-adjusted estimates and their precision (eg, 95% confidence interval). Make clear which confounders were adjusted for and why they were included **[Described under statistics (page 6-7) and results and tables pages 9-11]** |
|  |  | (*b*) Report category boundaries when continuous variables were categorized **[Reported under setting (page 5) and results (page 9-11) for altitudes. Rest N/A]** |
|  |  | (*c*) If relevant, consider translating estimates of relative risk into absolute risk for a meaningful time period **[N/A]** |
| Other analyses | 17 | Report other analyses done—eg analyses of subgroups and interactions, and sensitivity analyses |
| Discussion | | |
| Key results | 18 | Summarise key results with reference to study objectives **[Page 11]** |
| Limitations | 19 | Discuss limitations of the study, taking into account sources of potential bias or imprecision. Discuss both direction and magnitude of any potential bias **[Page 11]** |
| Interpretation | 20 | Give a cautious overall interpretation of results considering objectives, limitations, multiplicity of analyses, results from similar studies, and other relevant evidence **[Page 11, 14 and 15]** |
| Generalisability | 21 | Discuss the generalisability (external validity) of the study results **[Page 14-15]** |
| Other information | | |
| Funding | 22 | Give the source of funding and the role of the funders for the present study and, if applicable, for the original study on which the present article is based **[Given in acknowledgement section page 15, no specific funds were received]** |

*Give information separately for exposed and unexposed groups.

**Note:** An Explanation and Elaboration article discusses each checklist item and gives methodological background and published examples of transparent reporting. The STROBE checklist is best used in conjunction with this article (freely available on the Web sites of PLoS Medicine at http://www.plosmedicine.org/, Annals of Internal Medicine at http://www.annals.org/, and Epidemiology at http://www.epidem.com/). Information on the STROBE Initiative is available at www.strobe-statement.org.
